# Changes in the anteroposterior position of the femur relative to the tibia impact patient satisfaction in total knee arthroplasty

**DOI:** 10.1186/s12891-024-07679-5

**Published:** 2024-07-15

**Authors:** Tomofumi Kinoshita, Kazunori Hino, Tatsuhiko Kutsuna, Kunihiko Watamori, Hiroshi Kiyomatsu, Takashi Tsuda, Masaki Takao

**Affiliations:** Department of Orthopaedic Surgery, Ehime University Graduate School of Medicine, Ehime, Japan

**Keywords:** Total knee arthroplasty, Patient satisfaction, Anteroposterior position, Navigation system, Knee osteoarthritis

## Abstract

**Background:**

In this study, we aimed to investigate the preoperative and postoperative anteroposterior position (AP) of the femur relative to the tibia in total knee arthroplasty (TKA) and assess the influence of change in the AP position on clinical outcomes.

**Methods:**

We evaluated 49 knees that underwent bi-cruciate-substituted TKA using a navigation system. The preoperative and postoperative AP position of the femur relative to the tibia at maximum extension, 15°, 30°, 45°, 60°, 90°, 105°, and 120° and maximum flexion angles were calculated. The 2011 Knee Society Score was evaluated preoperatively and 1 year postoperatively. The Wilcoxon signed rank and Spearman’s rank correlation tests were performed, with statistical significance set at *P* < 0.05.

**Results:**

The postoperative AP position was significantly correlated with the preoperative AP position at each measured angle. The postoperative AP positions were statistically more anterior than those preoperatively. Furthermore, the changes in the AP position after TKA negatively correlated with the symptom (*P* = 0.027 at 30°, *P* = 0.0018 at 45°, *P* = 0.0003 at 60°, *P* = 0.01 at 90°, and *P* = 0.028 at 105°) and patient satisfaction (*P* = 0.018 at 60° and *P* = 0.009 at 90°) scores at 1 year postoperatively.

**Conclusion:**

The postoperative AP position of the femur relative to the tibia was strongly influenced by the preoperative those in TKA. Postoperative anterior deviation of the femur relative to the tibia from mid-flexion to deep flexion could worsen clinical outcomes.

## Background

Over the past decades, improving postoperative patient satisfaction following total knee arthroplasty (TKA) has been a significant challenge for knee surgeons and researchers [1]. Although several elements have been found to be critical factors in patient satisfaction [2], a perfect solution for achieving excellent results in all cases remains unknown. With progress in evaluation technology, assessing pre-, intra-, and postoperative knee status using various technical tools has become possible [3, 4]. Numerous studies have reported the influence of intraoperative elements on clinical outcomes [5, 6]. For instance, Nishio et al. reported that an intraoperative medial pivot motion improved postoperative patient satisfaction [5]. In addition, medial joint laxity and excessive tibial external rotation have been reported as unfavorable factors for clinical outcomes [6]. However, almost all previous studies have focused on the relationship between postoperative knee status and postoperative clinical results. Exploring changes in knee status and kinematics throughout TKA can present a solution to the problem of patient satisfaction after TKA.

Additionally, in almost all previous studies on knee kinematics, the central concern was the rotational kinematics of the medial pivot motion [5, 7, 8]. In addition to reporting normal knee rotational kinematics, some studies have proposed the occurrence of anterior paradoxical motion after TKA [9–11]. Moreover, the degree of preoperative varus deformity has been related to preoperative anterior paradoxical motion [12]. In these studies, the knees of many patients exhibited non-anatomical anteroposterior (AP) movement pre- and postoperatively that affected clinical outcomes. However, little attention has been paid to the AP kinematics and AP position. To restore normal knee kinematics, surgeons should be familiar with AP movement and understand changes in the AP knee position of the femur relative to the tibia in patients undergoing TKA.

In this study, we aimed to investigate the pre- and postoperative AP positions of the femur relative to the tibia using a navigation system and assess the influence of changes in the AP position of the femur relative to the tibia on clinical results. We hypothesized that the preoperative AP position of the femur could be related to the postoperative AP position of the femur and that postoperative fixation of the AP position of the femur would lead to better clinical results.

## Methods

This study was conducted in accordance with the Declaration of Helsinki and approved by the Institutional Review Board of Ehime University (identification number: 1,411,020). Additionally, written informed consent was obtained from all patients. This study evaluated 56 knees of 55 Japanese patients with osteoarthritis who underwent bicruciate-stabilized TKA (Journey II BCS: Smith & Nephew, London, UK). To accurately assess and minimize the influence of clinical variables, patients with preoperative flexion contracture > 15° (*n* = 5) and severe flexion restriction < 120° (*n* = 2) were excluded. The patient population comprised 43 female and 6 male, with a mean age of 75.9 ± 6.4 years (61–87 years). All patients presented with a varus deformity.

**Fig. 1:**
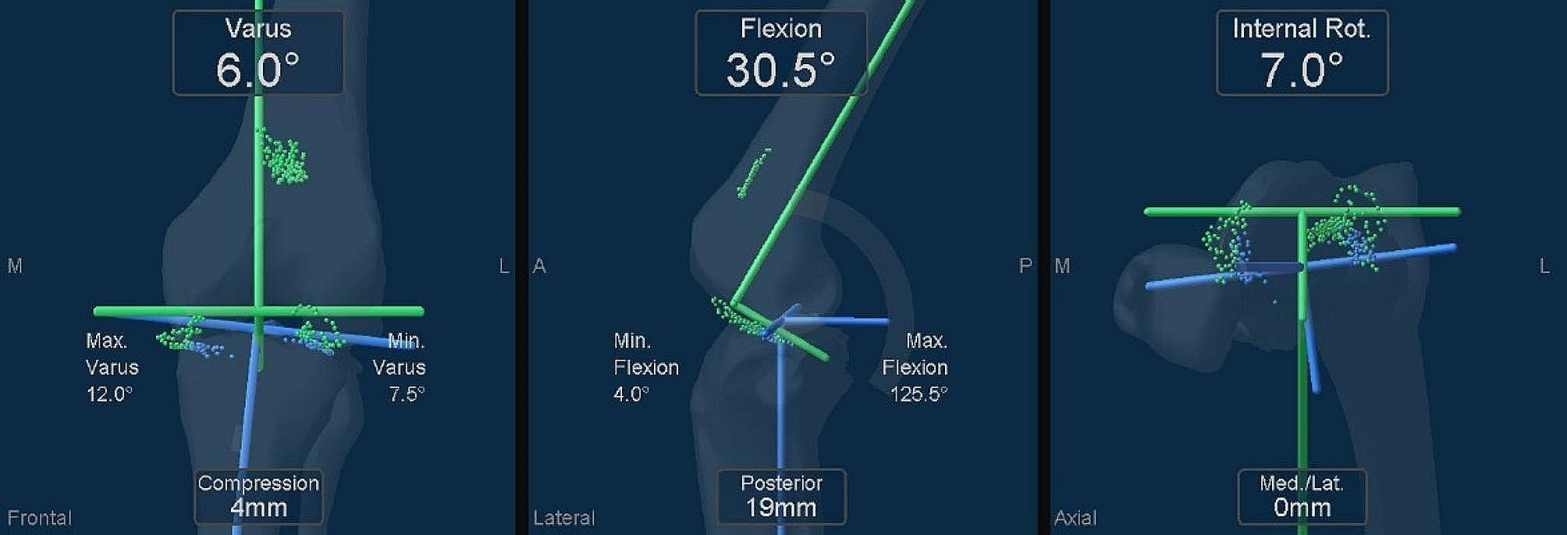
Measurement of the status of each knee using a navigation system. The left picture shows the varus-valgus and compression-distraction position of the tibia center relative to the femoral center. The middle picture shows the knee flexion angle and anteroposterior position of the tibia center relative to the femoral center. The right picture shows the rotational and medio-lateral position of the tibia center relative to the femoral center. Min, minimum; Max, maximum; Med, medial; Lat, lateral

**Fig. 2:**
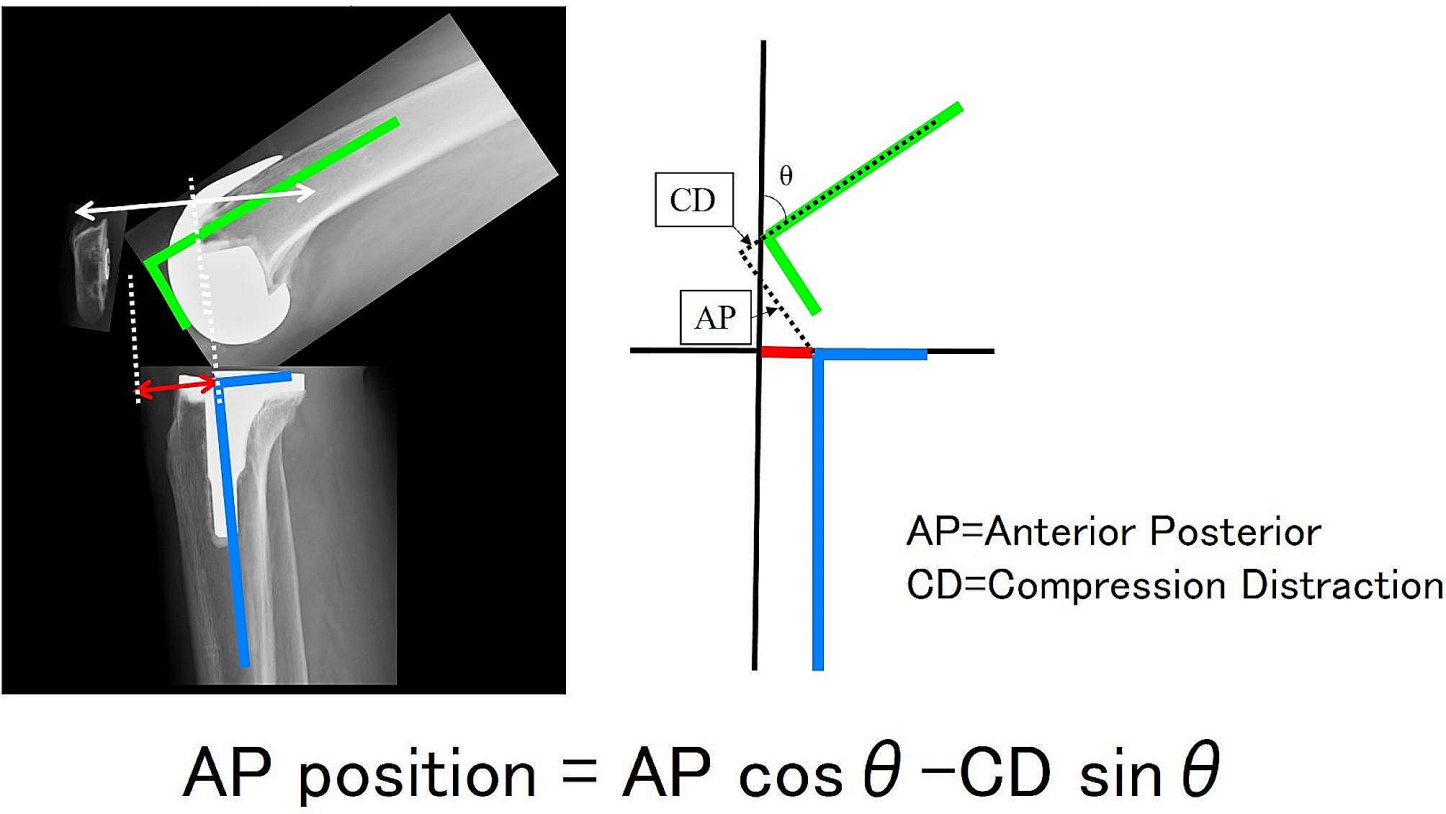
Measurement of the anteroposterior position of the femur relative to the tibia. The left picture shows the lateral view of the knee joint after bicruciate-stabilized total knee arthroplasty, and the right image shows a schematic representation of the navigation monitor evaluating the knee status of the same knee joint. The depicted equation was used to calculate the anteroposterior distance of the femoral center relative to the tibial center based on the parameters obtained from the navigation system. θ, knee flexion angle; AP, anterior-posterior distance of the tibial center relative to the femoral center; CD, distraction-compression distance of the tibial center relative to the femoral center

A navigation system (version 4.0, Precision Knee Navigation Software, Stryker, Kalamazoo, MI, USA) was used to evaluate the preoperative knee status. The air tourniquet was inflated to 250 mmHg when the patients were under general anesthesia. Furthermore, specific anatomical reference points were located by anchoring infrared signal transducers to the femur and tibia using pins. A midline skin incision was made to expose the subcutaneous tissue. Then, a knee joint was exposed using a medial parapatellar approach. Registration was performed using osteophytes and soft tissues, and the anterior cruciate ligament was preserved. The AP and rotational axes of the femur and tibia were identified based on the anatomical landmarks. In cases where it was difficult to determine the femoral axis due to deformity, Whiteside’s line was primarily used for the registration of the navigation system. The tibial rotational axis was set parallel to the line connecting one-third of the tibial tubercle to the center of the transverse diameter. After registration, the joint capsule was temporarily closed using four suture strands. Mild passive knee flexion was manually performed without angular acceleration while moving the leg from full extension to deep flexion. Then, the AP and compression-distraction status of the tibia center relative to the femur center at 0° (or maximum extension angle), 15°, 30°, 45°, 60°, 90°, 105°, and 120° and maximum flexion angles were automatically measured using the navigation system. Data were measured every 0.5° or 1 mm. Regarding the AP position of the femur relative to the tibia, we evaluated femoral center movement relative to the tibia as previously described [12, 13]. We calculated the AP position of the femur relative to the tibia using the status of the tibia relative to the femur obtained using a navigation system (Figs. 1 and 2). For the anteroposterior position, positive values indicated the anterior, whereas negative values represented the posterior position of the tibia relative to the femur. For the compression-distraction position, positive values indicated the compression, whereas negative values indicated the distraction position of the tibia relative to the femur. Therefore, the positive and negative signs of the AP and compression-distraction values changed depending on the position of the femur and tibia.

Subsequently, the distal femur was cut using a measured resection technique. To determine the rotational angle of the femoral component, we utilized the surgical transepicondylar axis as the index of femoral rotation. Before the surgery, we calculated the angle gap between the surgical transepicondylar axis and the posterior condylar axis on the axial view of computed tomography to determine the rotational angle. Concerning bone resections, the distal femoral cut was made perpendicular to the mechanical axis of the femur, and the proximal tibial cut was made perpendicular to the mechanical axis of the tibia based on the concept of mechanical alignment. The posterior tibial slope was set at 3° in all cases in this study. After removing the osteophytes, we placed trial components and a trial insert. We typically began with a 9-mm insert (the thinnest insert thickness in this TKA procedure). Then, we evaluated the knee stability using the manual varus–valgus test throughout extension to deep flexion in that condition. Finally, we performed the POLO test to confirm the stability in the 90° flexion position [14]. We increased the size of the insert in cases showing excessive medial laxity and excessive extension and flexion laxity. Conversely, in cases that showed flexion contracture or inappropriate soft-tissue balance, the posterior knee capsule, medial collateral ligament, or other tissues were carefully and selectively released to achieve intraoperative full extension and correct soft-tissue balance throughout the range of motion [15]. After the trial, the components and inserts of the proper thickness were placed in the appropriate position with cement. Thereafter, the surgical incision was closed. Subsequently, we assessed the AP position of the femur relative to the tibia using the status of the tibia relative to the femur obtained by a navigation system, similar to procedures performed before TKA. The same surgeon performed all surgeries.

The test–retest reliability of each status obtained using the navigation system was calculated to confirm the accuracy of measurements. The test–retest reliability of the AP and compression-distraction status was evaluated, yielding sufficiently high interclass and intraclass correlation coefficients (> 0.9 at each measured angle of knee flexion). In addition, the range of motion of the knee joint was assessed preoperatively and 1 year postoperatively. The 2011 Knee Society Score (KSS) was used to evaluate clinical outcomes [16]. This questionnaire was used for all patients preoperatively and 1 year postoperatively. For radiographic evaluation, PTS was evaluated preoperatively and 1 year postoperatively using short knee radiographs. The PTS was measured using short knee lateral radiographs and evaluated as the angle between the medial tibial plateau and the posterior cortical line of the proximal tibia. The changes in patient characteristics are presented in Table 1.

**Table 1:** Pre- and postoperative patient characteristics

		Preoperative	Postoperative	*P*-value
		Mean ± SD	Mean ± SD	
HKA-angle		11.6 ± 5.2°	0.8 ± 2.5°	< 0.0001*
MPTA		83.8 ± 2.9°	89.3 ± 1.7°	< 0.0001*
LDFA		89.7 ± 3.0°	88.3 ± 2.2°	0.0019*
Posterior tibial slope		8.8 ± 2.9°	3.0 ± 2.4°	< 0.0001*
Maximum extension angle		10.0 ± 7.0°	0.6 ± 2.0°	< 0.0001*
Maximum flexion angle		119.9 ± 14.4°	128.0 ± 11.7°	< 0.0001*
KSS	Symptoms	7.6 ± 5.2	20.1 ± 4.2°	< 0.0001*
	Patient satisfaction	12.9 ± 6.7	28.5 ± 7.2°	< 0.0001*
	Patient expectation	13.8 ± 2.0	11.6 ± 2.4°	< 0.0001*
	Functional activity	35.2 ± 17.4	62.3 ± 16.7°	< 0.0001*

SD, standard deviation; HKA, hip-knee-ankle; LDFA, lateral distal femoral angle; MPTA, medial proximal tibial angle; KSS, 2011 Knee Society Score

* *P* < 0.05

### Statistical analysis

Statistical analyses were performed using JMP (version 14.0, SAS Institute, Tokyo, Japan). Non-parametric tests were performed in this study because the data were found to be non-normally distributed using the Shapiro–Wilk test. The non-parametric Wilcoxon signed-rank test was performed to determine the differences between the anteroposterior position of the femur relative to the tibia before and after TKA. Spearman’s rank correlation coefficient (ρ) was used to evaluate the relationship among the AP position of the femur relative to the tibia, PTS, and KSS. A power analysis was conducted based on the mean and standard deviation calculated from three preliminary consecutive measurements. The required minimum sample size of 34 was determined to achieve a correlation of δ = 5 and σ = 5, with 80% power and α = 0.05, accounting for the results of the mean difference in AP position of the femur relative to the tibia before and after TKA. Accordingly, we assessed 49 participants to compensate for the small sample size in this study. Statistical significance was set at a P value of < 0.05.

## Results

### AP position of the femur relative to the tibia

**Fig. 3:**
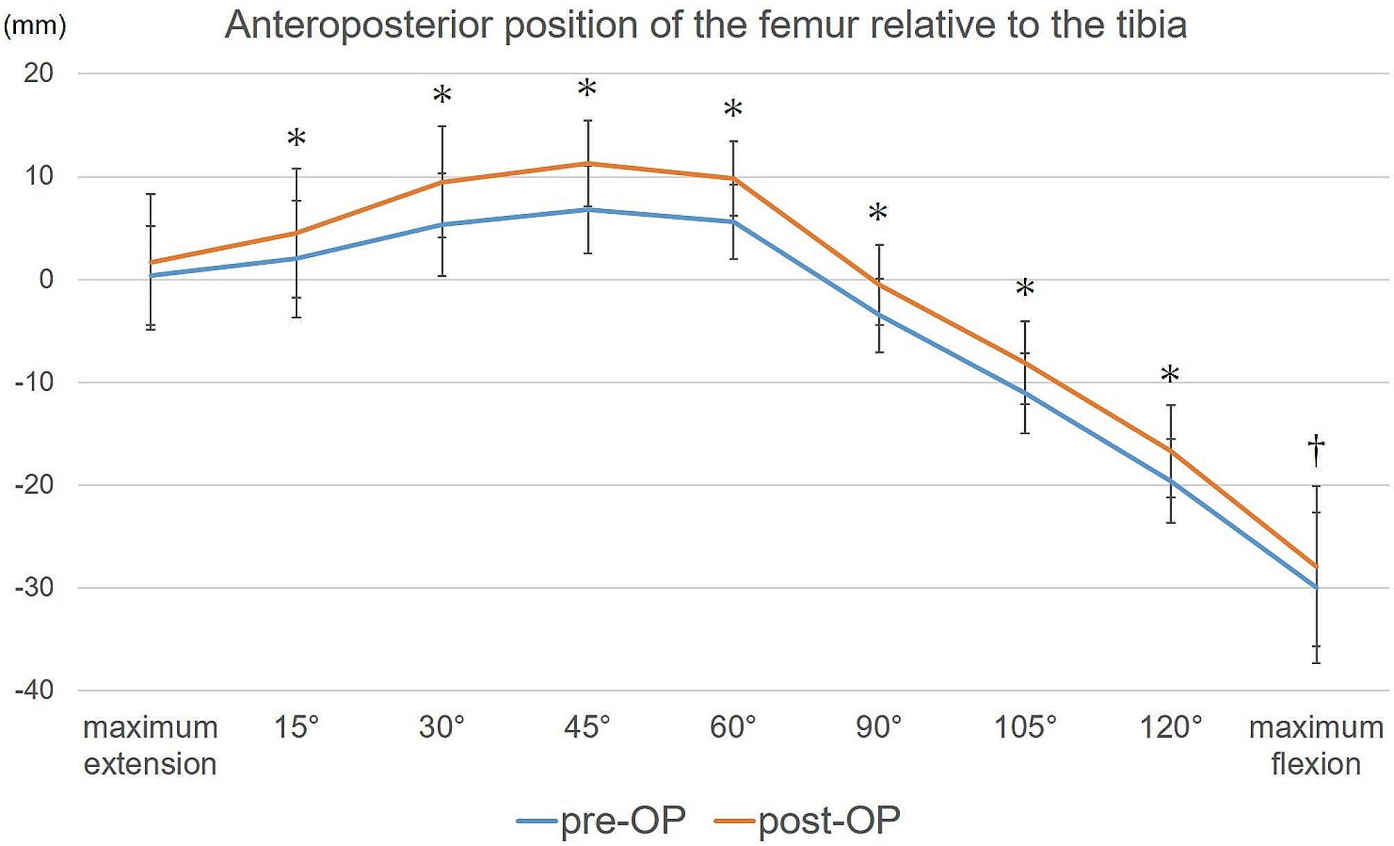
Preoperative and postoperative anteroposterior position of the femur relative to the tibia. The mean anteroposterior position of the femur relative to the tibia at each knee flexion angle. The graph shows changes in the anteroposterior position of the femur relative to the tibia throughout the range of motion. The horizontal line shows the knee flexion angle, and the vertical line shows the anteroposterior position of the femur relative to the tibia (a positive value indicates the anterior position of the femur relative to the tibia). Asterisks: *P* < 0.01; dagger: *P* < 0.05. AP, anteroposterior position; pre-OP, preoperative; post-OP, postoperative; maximum extension, maximum knee extension angle; maximum flexion, maximum knee flexion angle

The postoperative AP position of the femur relative to the tibia correlated with the preoperative AP position of the femur relative to the tibia at each measured angle (Table 2). Tables 3 and 4 show the relationship between the AP position of the femur relative to the tibia and PTS. The changes in PTS after TKA did not correlate with those in the AP position of the femur relative to the tibia, except during extension to early knee flexion (Table 5). Figure 3 shows the preoperative and postoperative AP position of the femur relative to the tibia throughout the range of motion. The postoperative AP positions of the femur relative to the tibia at all measured angles were statistically more anterior than they were preoperatively (ρ = 0.46, 0.47, 0.61, 0.58, 0.46, 0.41, 0.35, 0.36, 0.64 at maximum extension, 15°, 30°, 45°, 60°, 90°, 105°, 120° and maximum flexion, respectively).

**Table 2:** Correlation coefficients between preoperative and postoperative AP positions at each angle

	Preoperative	Postoperative	ρ	*P*-value
	Mean ± SD	Mean ± SD		
Maximum knee extension angle	0.4 ± 6.6	1.7 ± 4.8	0.46	0.0009**
15°	2.0 ± 6.3	4.5 ± 5.7	0.47	0.0008**
30°	5.3 ± 5.4	9.5 ± 5.0	0.61	< 0.0001**
45°	6.8 ± 4.2	11.3 ± 4.3	0.58	< 0.0001**
60°	5.6 ± 3.6	9.8 ± 3.6	0.46	0.0009**
90°	-3.5 ± 3.9	-0.5 ± 3.6	0.41	0.0038**
105°	-11.1 ± 4.0	-8.1 ± 3.9	0.35	0.016*
120°	-19.6 ± 4.5	-16.7 ± 4.1	0.36	0.011*
Maximum knee flexion angle	-30.0 ± 7.8	-27.9 ± 7.3	0.64	< 0.0001**

SD, standard deviation; AP position, the anteroposterior position of the femur relative to the tibia

* *P* < 0.05; ** *P* < 0.01

**Table 3:** Correlation coefficients between the preoperative posterior tibial slope and AP position at each angle

	Preoperative AP position of the femur at each angle	ρ	*P*-value
Preoperative posterior tibial slope	Maximum knee extension angle	-0.48	0.0004**
	15°	-0.43	0.0018**
	30°	-0.34	0.014*
	45°	-0.15	n.s.
	60°	-0.06	n.s.
	90°	-0.03	n.s.
	105°	0.02	n.s.
	120°	0.01	n.s.
	Maximum knee flexion angle	0.29	0.038*

AP position, anteroposterior position of the femur relative to the tibia; n.s., non-significant

* *P* < 0.05; ** *P* < 0.01

**Table 4:** Correlation coefficients between the postoperative posterior tibial slope and AP position at each angle

	Postoperative AP position of the femur at each angle	ρ	*P*-value
Postoperative posterior tibial slope	Maximum knee extension angle	-0.43	0.0018**
	15°	-0.30	0.034*
	30°	-0.39	0.0054**
	45°	-0.38	0.0065*
	60°	-0.32	0.023*
	90°	-0.27	n.s.
	105°	-0.28	0.04*
	120°	-0.10	n.s.
	Maximum knee flexion angle	-0.05	n.s.

AP position, anteroposterior position of the femur relative to the tibia; n.s., non-significant

* *P* < 0.05; ** *P* < 0.01

**Table 5:** Correlation coefficients between the changes in postoperative posterior tibial slope and the changes in the AP position at each angle

	Changes in the AP position of the femur at each angle	ρ	*P*-value
Changes in PTS	Maximum knee extension angle	-0.56	< 0.0001**
	15°	-0.46	0.0009**
	30°	-0.35	0.012*
	45°	-0.15	0.28
	60°	-0.18	0.21
	90°	-0.13	0.34
	105°	-0.08	0.55
	120°	-0.02	0.87
	Maximum knee flexion angle	0.04	0,73

PTS, posterior tibial slope; AP position: anteroposterior position of the femur relative to the tibia

*, *p* < 0.05; **, *p* < 0.01

### Clinical outcomes

**Fig. 4:**
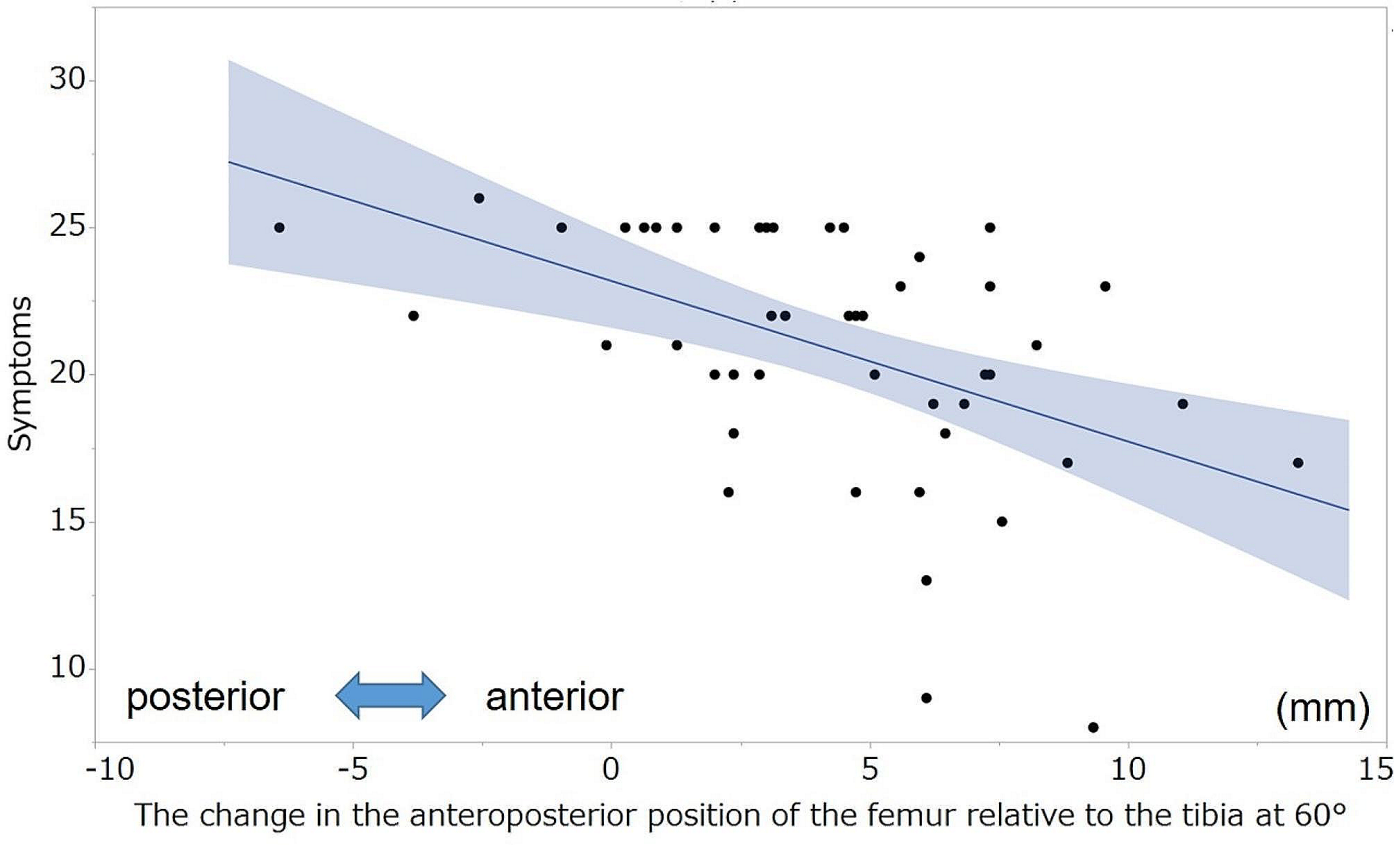
Correlation between the symptom score and the change in the anteroposterior position of the femur at 60°. The graph shows the scatterplots of the symptom scores of the KSS and changes in the anteroposterior position of the femur relative to the tibia at 60°. Symptoms, symptom score of KSS; KSS, 2011 Knee Society Score

**Fig. 5:**
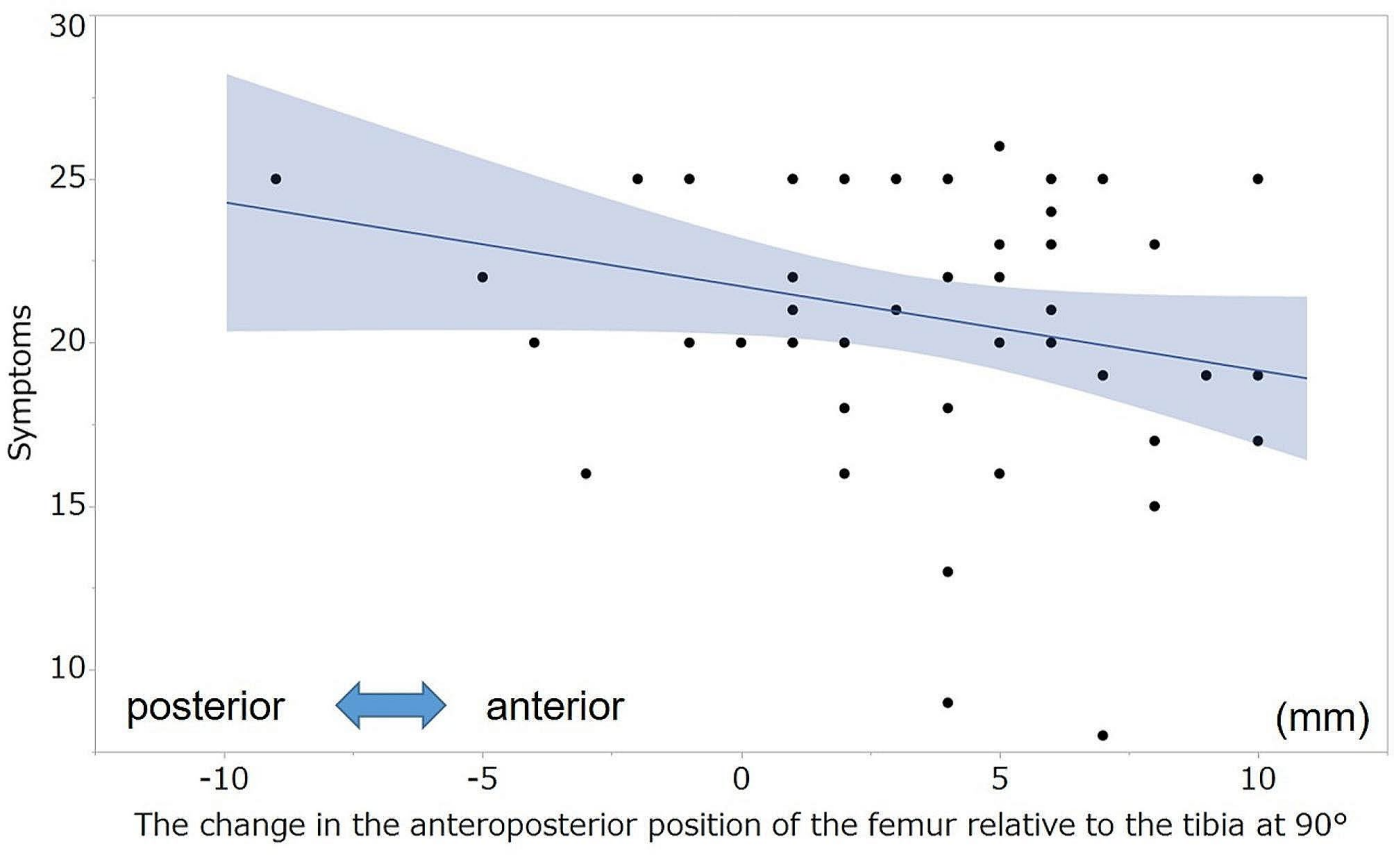
Correlation between the symptom score and the anteroposterior position of the femur at 90°. The graph shows scatterplots of the symptom score of the KSS and the change in the anteroposterior position of the femur relative to the tibia at 90°. Symptoms, symptom score of KSS; KSS, 2011 Knee Society Score

**Fig. 6:**
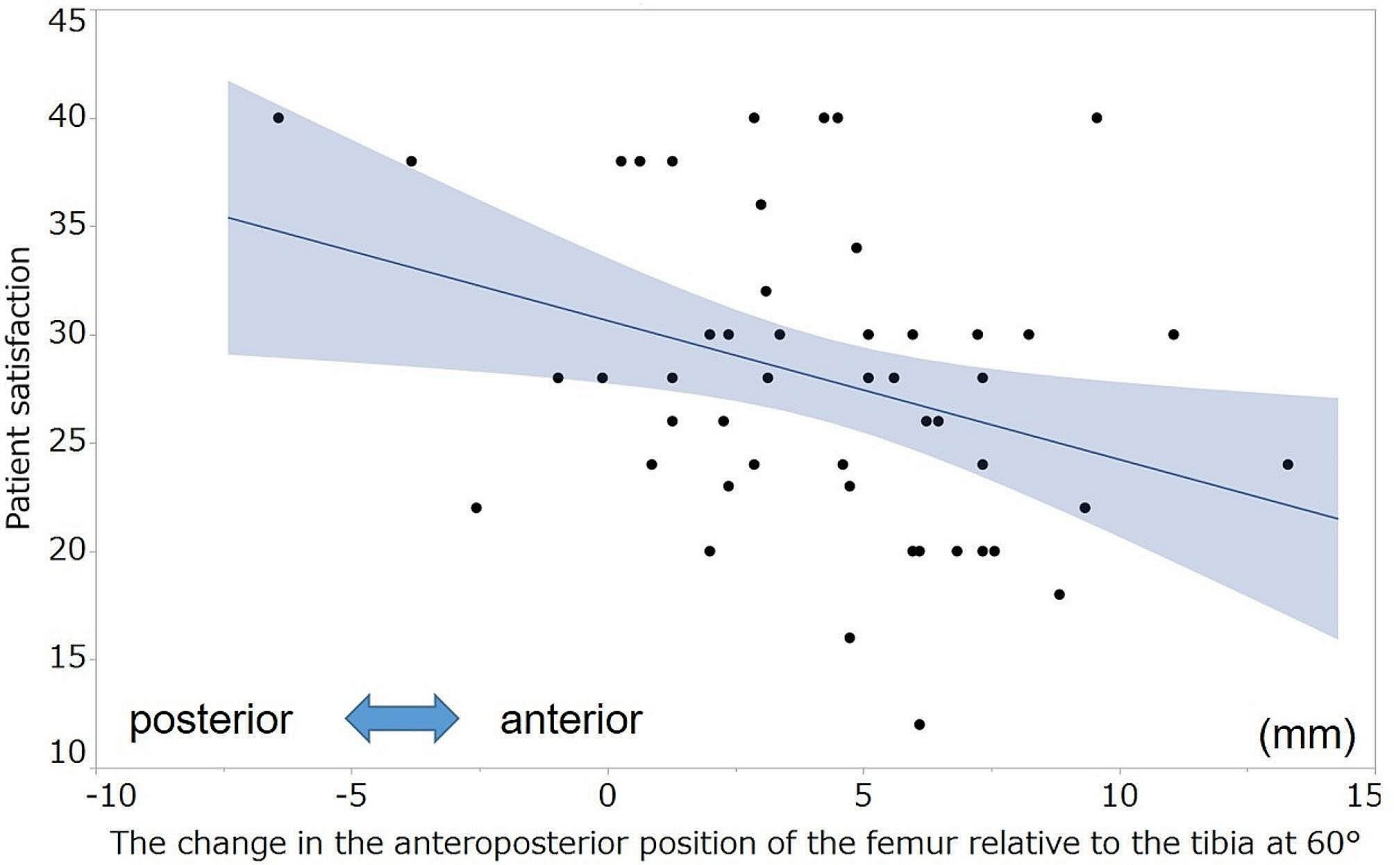
Correlation between patient satisfaction and the change in the anteroposterior position of the femur at 60°. The graph shows the scatter plots of the patient satisfaction score of the KSS and the change in the anteroposterior position of the femur relative to that of the tibia at 60°. Patient satisfaction, patient satisfaction score of KSS; KSS, 2011 Knee Society Score

**Fig. 7:**
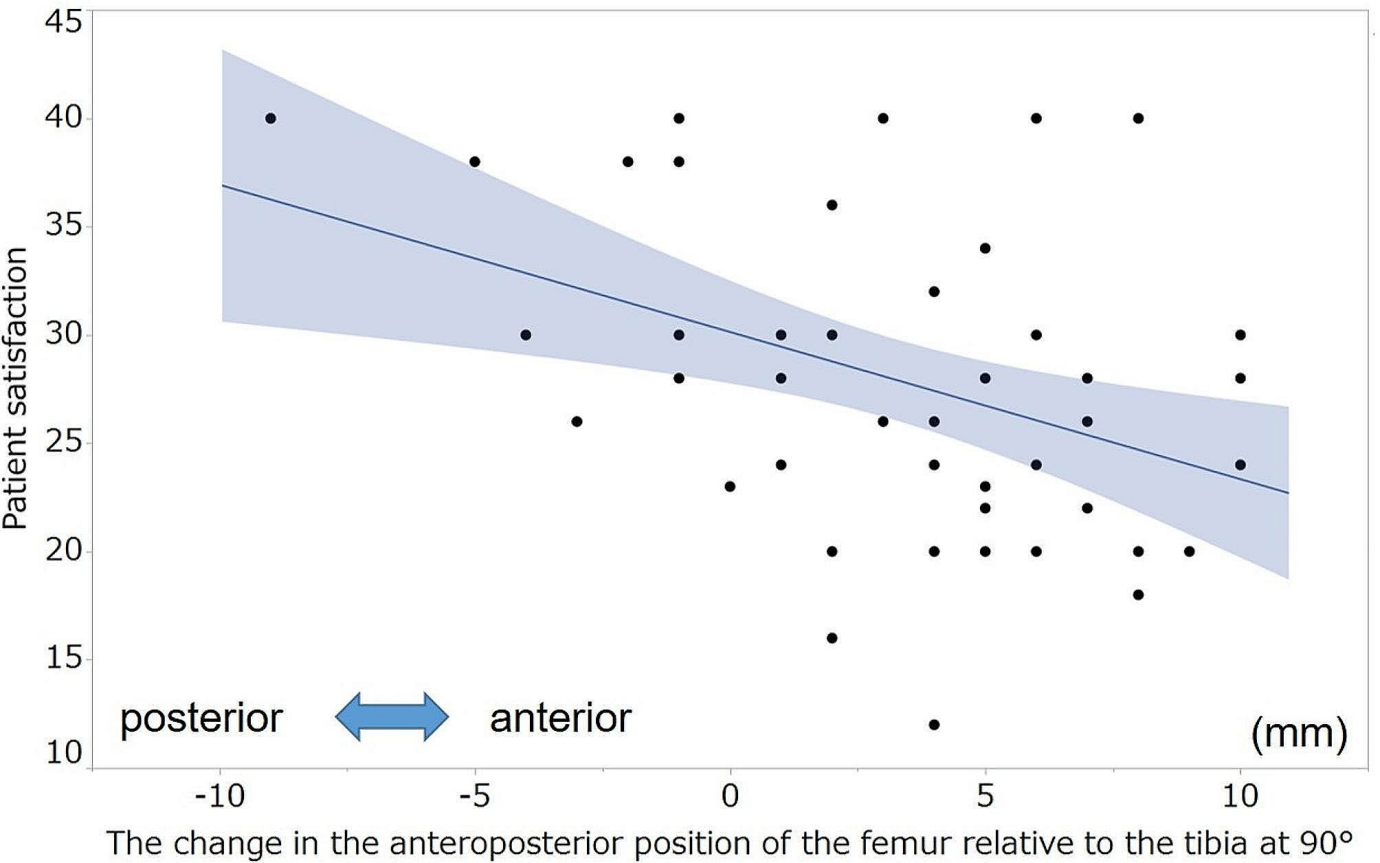
Correlation between the satisfaction score and the change in the anteroposterior position of the femur at 90°. The graph shows the scatter plots of the patient satisfaction score of the KSS and the change in the anteroposterior position of the femur relative to that of the tibia at 90°. Patient satisfaction, patient satisfaction score of KSS; KSS, 2011 Knee Society Score

No significant correlation was observed between the KSS and postoperative AP position of the femur relative to the tibia at each measured angle. Table 6 summarizes the correlation coefficients between the KSS and change in the AP position of the femur relative to the tibia. The postoperative changes in the AP position of the femur relative to the tibia at 30°,45°, 60°, 90°, and 105° were negatively correlated with the symptom score (ρ=-0.33, -0.46, -0.52, -0.38, -0.33, respectively; Figs. 4 and 5). Moreover, the postoperative change in the AP position of the femur relative to the tibia at 60° and 90° was negatively correlated with the patient satisfaction score (ρ=-0.35, -0.39, respectively; Figs. 6 and 7). However, no statistically significant correlation was observed between the KSS and change in the PTS after TKA.

**Table 6:** Correlation coefficients between changes in the AP position and KSS

Change in the AP position at each angle	Symptoms		Patient satisfaction		Patient expectation		Functional activity	
	ρ	*P*	ρ	*P*	ρ	*P*	ρ	*P*
Maximum knee extension	-0.11	n.s.	0.003	n.s.	-0.17	n.s.	-0.25	n.s.
15°	-0.10	n.s.	0.06	n.s.	0.01	n.s.	-0.01	n.s.
30°	-0.33	0.027*	-0.07	n.s.	-0.18	n.s.	-0.09	n.s.
45°	-0.46	0.0018**	-0.25	n.s.	-0.21	n.s.	-0.06	n.s.
60°	-0.52	0.0003**	-0.35	0.018*	-0.30	0.04*	-0.10	n.s.
90°	-0.38	0.01*	-0.39	0.009**	-0.25	n.s.	0.03	n.s.
105°	-0.33	0.028*	-0.21	n.s.	-0.11	n.s.	0.15	n.s.
120°	-0.24	n.s.	-0.26	n.s.	-0.08	n.s.	0.18	n.s.
Maximum knee flexion	-0.03	n.s.	-0.10	n.s.	0.11	n.s.	-0.13	n.s.

AP position, anterior-posterior position of the femur relative to the tibia; KSS, The 2011 Knee Society Score; Symptoms, symptom score for the KSS; Patient satisfaction, patient satisfaction score for the KSS; Patient expectation, patient expectation score for the KSS; Functional activity, functional activity score for the KSS; Maximum knee extension, maximum knee extension angle; Maximum knee flexion, maximum knee flexion angle; n.s., non-significant

* *P* < 0.05; ** *P* < 0.01

## Discussion

The most important finding of this study was that the change in AP position of the femur relative to the tibia was associated with the clinical results after TKA. Postoperative anterior deviation of the femur relative to the tibia during mid-flexion led to unfavorable clinical outcomes. Moreover, the preoperative AP position of the femur relative to the tibia was strongly related to the postoperative AP position of the femur relative to the tibia. To the best of our knowledge, this is the first study to demonstrate that a change in the AP position of the femur relative to the tibia impacts patient satisfaction after TKA. These results may aid in alleviating persistent issues regarding TKA.

Previous studies on the AP knee status focused on three factors: position, kinematics, and stability [17–27]. A previous study demonstrated the influence of the implant design on the AP position of the femur relative to the tibia [14]. The unique implant design in patients undergoing BCS-TKA induced proper positioning of the femur, resulting in a lower offset ratio closer to that of the normal knee, at knee extension [17]. Another study demonstrated the relationship between the intraoperative factor and the AP position of the femur and found that the PTS was correlated with the AP position of the femur in patients undergoing TKA with cruciate-substituting inserts, but not in those undergoing TKA with cruciate-retaining inserts [18]. In the present study, which utilized BCS-TKA, the AP position of the femur relative to the tibia during mid-flexion was negatively correlated with the PTS before and after TKA. In addition, postoperative anterior deviation of the femur during knee flexion has been shown to lead to poor clinical outcomes. Thus, drastic postoperative changes in the AP position of the femur relative to the tibia should be avoided in TKA. However, further validation is needed to establish a technique to surgically control the AP position, which varies from case to case preoperatively.

Studies on AP kinematics [19–21] have demonstrated the non-physiological anterior femoral movement during knee flexion after TKA. Moreover, in knees with osteoarthritis, the degree of deformity has been shown to contribute greatly to such an anterior paradoxical motion in knees with preoperative osteoarthritis [3]. Such non-physiological anterior femoral movement impacts the postoperative clinical outcomes [22]. Sakai et al. researched the influence of the AP position of the femur and AP kinematics in cruciate retaining TKA, and demonstrated that anterior position of the femur during mid-flexion correlated with postoperative functional activities score [23]. These results might be derived from PCL tension and patella-femoral pressure. Konno et al. demonstrated the normal knee rotational kinematics reduced the patella-femoral pressure [24]. From the point of view, the restore of the normal knee kinematics after TKA has a possibility to resolve these problems including our results. Furthermore, postoperative AP stability in TKA has been reported in previous studies [25–29]. Mochizuki et al. reported that excessive postoperative AP instability at mid-flexion directly led to anxiety during daily movement [26]. Although the influence of AP kinematics and AP stability on clinical results was not assessed in the present study, changes in the AP position of the femur relative to the tibia before and after surgery were observed to be related to the pain and satisfaction scores after TKA. This may be attributed to the soft tissue strain due to drastic changes in the AP position. In this study, the preoperative AP position of the femur was correlated with postoperative AP positions of the femur. The results of this study suggest that surgeons should pay attention to the preoperative AP position, which varies considerably across patients. To address various AP factors such as position, stability, and kinematics, further research is needed to determine the appropriate surgical method to avoid anterior deviation of the femur relative to the tibia.

This study has some limitations. First, the evaluation was not performed in a weight-bearing state due to intraoperative evaluation. Although knee kinematics have been reported to show the same pattern under weight-bearing and non-weight-bearing conditions [30], further research is required. Second, the preoperative conditions of cartilage wear, the anterior cruciate ligament, and the posterior cruciate ligament, which may influence the AP position of the femur relative to the tibia, were not investigated. Third, this study did not clarify the specific surgical method tailored according to patients’ conditions. These limitations may restrict the generalizability of the results of this study. However, we propose that the change in the AP position of the femur relative to the tibia might have a predominant effect on clinical results in patients undergoing TKA.

## Conclusions

The postoperative AP position of the femur relative to the tibia was strongly influenced by the preoperative AP position of the femur relative to the tibia in TKA. Postoperative anterior deviation of the femur relative to the tibia from mid-flexion to deep flexion could worsen clinical outcomes.

## Data Availability

The datasets used and/or analysed during the current study available from the corresponding author on reasonable request.

## Abbreviations

AP: Anteroposterior
KSS: Knee Society Score
PTS: Posterior tibial slope
TKA: Total knee arthroplasty
BCS: Bicruciate-stabilized
